# Fear of fertility side effects is a major cause for COVID-19 vaccine hesitance in infertile patients

**DOI:** 10.3389/fmed.2023.1178872

**Published:** 2023-06-01

**Authors:** Jessica Kern, Cordula Schippert, Delnaz Fard, Alexandra Petra Bielfeld, Frauke von Versen-Höynck

**Affiliations:** ^1^Department of Obstetrics and Gynecology, Division of Reproductive Medicine, Hannover Medical School, Hannover, Germany; ^2^Department of Obstetrics, Gynecology and Reproductive Endocrinology and Infertility, UniKiD/UniCareD, Düsseldorf, Germany

**Keywords:** COVID-19, vaccination, vaccine hesitance, fertility treatment, infertility

## Abstract

**Introduction:**

This study aims to investigate the acceptance, hesitance and attitudes of infertile female patients toward the COVID-19 vaccination.

**Methods:**

An anonymous cross-sectional online survey was conducted between 28th of January to 10th of August 2022. The questionnaire consisted of 35 questions on demographics, COVID-19 vaccination status, prior concerns of the vaccinated participants and reasons for not vaccinating among unvaccinated participants, and factors influencing the decision not to vaccinate.

**Results:**

Of 406 participants who answered all questions, 92.1% reported having received at least one dose of COVID-19 vaccine, 7.9% were unvaccinated. Factors associated with the decision for vaccination were full time or part time employment (*p* = 0.05), high trust in the principle of vaccination (*p* < 0.001), high willingness for other vaccination during fertility treatment (p < 0.001) and risk factors for severe COVID-19 (*p* = 0.007). Concerns about directly occurring adverse effects after vaccination (42.0%), about impact on own fertility (21.9%) or on the fertility treatment (27.5%) were the main concerns beforehand of vaccinated participants. Correlations between fertility concerns and mistrust in the general principle of vaccination were found. Beside general health concerns, unvaccinated participants reported fears about fertility impairment as the most important arguments against a COVID-19 vaccination (median of 5.0 on a five-point-Likert scale).

**Conclusion:**

Both vaccinated and unvaccinated participants stated having concerns and fears about side effects of the COVID-19 vaccination on their fertility. To increase patients’ trust in medical recommendations, such as vaccination, to avoid mistrust in the medical system and to maintain patient’s compliance, there should be additional educational services that address infertile patients and their needs.

## 1. Introduction

Since the WHO declared the outbreak of the novel coronavirus SARS-CoV-2 a pandemic on 11 March 2020, there have been profound changes in health care policy for both medical personnel and patients (1).

In addition to numerous recommendations regarding contact reduction and the closure of public places, most fertility clinics were closed for several weeks following the advice of international [e.g., European Society of Human Reproduction and Embryology (ESHRE)] and national [e.g., German Society for Reproductive Medicine (DGRM)] societies (2). This led to a high dissatisfaction and additional emotional stress among patients undergoing infertility treatment (3).

The introduction of vaccines in late December 2020 led to a reduction in the incidence of severe courses of the disease (4), which marked a key step in pandemic control. In September 2021, the German Vaccination Commission (*Ständige Impfkommission, STIKO*) subsequently issued recommendations for vaccination against SARS-CoV-2 from the second trimester of pregnancy onwards (5). Additionally, women who intended to become pregnant were advised to get vaccinated before conception (5) because pregnancy itself was recognized as a risk factor for severe COVID-19 with adverse maternal and fetal outcomes (6). Despite these recommendations, the acceptance rate for vaccination against SARS-CoV-2 in the group of pregnant women was overall lower than in the rest of the population (7–10). Still, various fears and concerns about SARS-CoV-2 vaccination including a negative effect on fertility (11) continue to emerge in daily practice, which requires increased educational efforts.

Most of the studies published to date shed light on the attitude of pregnant or breastfeeding patients (7–10) and little is known about the vaccination status, attitudes and concerns among patients planning to conceive, e.g., those undergoing fertility treatment. Previous studies reported hesitant attitudes of infertile couples toward vaccination (12) but did not illuminate in detail the reasons to decide against vaccination. However, since pregnancy itself is a risk factor for a severe course of COVID-19 (6), women already intending to become pregnant should also be persuaded to be vaccinated.

The primary aim of the study is to draw conclusions about the COVID-19 vaccination status of women seeking treatment in fertility clinics, and to investigate their attitude toward vaccination, their fears and concerns.

These new findings will help to comprehensively counsel patients and couples who wish to conceive and to adequately address patient’s wishes and concerns in exceptional situations, e.g., the COVID-19 pandemic.

## 2. Methods

### 2.1. Participants

After obtaining ethical approval from the Ethics Committee of Hannover Medical School (approval no.: 10174_BO_K_2022) women seeking fertility treatment were asked to participate in an online survey. Recruitment took place in two ways. On the one hand, participants were directly informed about the survey by staff or by flyers, which were distributed among fertility clinics in Germany. In addition, the link to the survey was posted on online platforms and three times in Facebook groups for women with an unfulfilled desire to conceive. The survey was available online from 28th January to 10th August 2022.

To proceed with the questionnaire, in the beginning participants had to give their informed consent online. Otherwise, they were led to the end of the survey without being able to answer it. To be included in the analysis, participants had to answer every question and finish the survey.

### 2.2. Questionnaire

The cross-sectional, anonymous online survey was designed in German language by a team of three reproductive medicine specialists and a medical student. Professional knowledge and a systematic literature review were used to develop the survey on the SoSciSurvey platform. The survey was piloted by physicians of two academic fertility centers and by persons who were of reproductive age without a medical background. The questionnaire was revised based on their replies and comments and contained a total of 35 questions. A translated version can be found in Supplementary material. The survey began with questions about demographic information, fertility and pregnancy history. After that, participants were asked about basic trust in vaccination against other diseases. This first part ended with the question about the vaccination status. Afterwards the survey was split into those vaccinated or not vaccinated. Vaccinated participants were asked about reasons for the decision to receive a COVID-19 vaccination and possible concerns beforehand. The unvaccinated population had to rate various reasons against vaccination using a five-point Likert scale (from 1 = I do not agree at all to 5 = I totally agree) to allow for a more complex ranking of the statements and to capture finer differences. The last part included additional questions about factors having had an influence on the decision not to get vaccinated.

### 2.3. Data analysis

First, basic frequencies of every question were calculated for both vaccinated and unvaccinated participants. Variables, e.g., age or educational status were summarized in groups and are shown as mean with standard deviations (SD). After univariate analysis, correlation between demographic data and vaccination status were investigated using Pearson Chi square test for homogeneity for categories variables. To detect differences in continuous variables, the Student’s t-test was applied. For the vaccinated group, correlations between general trust in vaccination, the duration and emotional impact of the fertility treatment and concerns before COVID-19 vaccination were analyzed. For this purpose, odds ratios and their 95 per cent confidence intervals were calculated. For the unvaccinated group, answers including a 5-point Likert scale were analyzed by calculating the median and the interquartile range. All tests were two-sided and considered statistically significant at a value of *p* of < 0.05.

Data analysis was performed using IBM SPSS 27 for statistical analysis.

## 3. Results

### 3.1. Demographics and SARS-CoV-2 vaccination status

A total of 981 people clicked on the link while 485 participants answered at least one question of the survey, giving a response rate of 49.4%. In the end, 406 women seeking fertility treatment answered the survey completely and their data were included in the final analysis.

Detailed demographic information of the study population sorted by vaccination status can be seen in Table 1. The average age was similar for vaccinated and unvaccinated participants (34.5 ± 4.37 and 34.26 ± 4.60 years, *p* = 0.78). The population was highly educated with 50.0% of the unvaccinated and 60.7% of the vaccinated individuals having completed tertiary education (*p* = 0.24). Most of them were employed full time or part time (81.3% vs. 89.0%, *p* = 0.05). Participants from all German federal states were represented, with the majority (30.8%) from Lower Saxony. The detailed distribution can be found in Supplementary Table 1. A secondary sterility with at least having one pregnancy in the past was reported by 210 women while 148 of the participants reported at least one miscarriage.

**Table 1:** Demographic characteristics sorted by vaccination status.

Characteristic	Unvaccinated (*N* = 32)	Vaccinated (*N* = 374)	*p*-value
**General information**			
Age	34.50 ± 4.37	34.26 ± 4.60	0.78
Highest level of education completed			0.24
Secondary education	16 (50.0)	147 (39.3)	
Tertiary education	16 (50.0)	227 (60.7)	
Current status of employment			0.05
Employed full time or part time	24 (75.0)	333 (89.0)	
Self-employed	2 (6.3)	13 (3.5)	
Still in education	0	5.1 (1.3)	
On parental leave	4 (12.5)	16 (4.3)	
Unemployed/Job-seeking	1 (3.1)	1 (0.3)	
Disability/on leave	1 (3.1)	6 (1.6)	
**Pregnancy and fertility treatment**			
(for the 210 women who reported at least one pregnancy in the past)			
Number of given life births			0.13
0	5 (15.6)	111 (29.7)	
1	8 (25.0)	71 (19.0)	
2	1 (3.1)	10 (2.7)	
3 or higher	0	4 (1.1)	
Number of miscarriages			0.06
0	5 (15.6)	57 (15.3)	
1	8 (25.0)	78 (20.9)	
2	0	30 (8.0)	
3	1 (3.1)	25 (6.7)	
4 or higher	0	6 (2.9)	
Number of induced abortions			0.39
0	14 (43.8)	186 (49.5)	
1	0	10 (2.7)	
Number of ectopic pregnancies			0.92
0	13 (40.6)	176 (47.1)	
1	1 (3.1)	19 (5.1)	
2	0	1 (0.3)	
Length of time trying to conceive			0.41
<1 year	3 (9.4)	23 (6.1)	
More than 1 less than 2 years	5 (15.6)	97 (25.9)	
More than 2 less than 5 years	19 (59.4)	184 (49.2)	
>5 years	5 (15.6)	70 (18.7)	
Mental distress through the unfulfilled wish to conceive and fertility treatments			0.26
Very high	9 (28.1)	143 (38.2)	
A little high	15 (46.9)	122 (32.6)	
Neutral	5 (15.6)	95 (25.1)	
Rather low	3 (9.4)	15 (4.0)	
Very low	0	0	
Treatments received so far			0.86
None	1 (3.1)	14 (3.7)	
Timed intercourse	21 (65.6)	275 (73.5)	
Intrauterine insemination	12 (37.5)	104 (27.8)	
IVF/ICSI	23 (71.9)	220 (58.8)	
Frozen embryo transfer cycle	10 (31.3)	100 (26.7)	
Other	1 (3.1)	21 (5.6)	
**COVID-19 experience**			
Friend or a close relative who has or has had severe COVID-19	8 (12.5)	101 (27.0)	0.81
Loss of friend or close family member to COVID-19	3 (9.4)	38 (10.2)	0.89
High COVID-19 exposure in the workfield (e.g., heath care).	5 (15.6)	87 (23.3)	0.32
Risk factors for severe COVID-19			0.01
No risk factors	27 (84.4)	232 (62.0)	
Obesity	3 (9.4)	82 (21.9)	
Diseases of the airways	1 (3.1)	30 (8.0)	
Autoimmune disease	3 (9.4)	42 (11.2)	
Other	0	39 (10.4)	
Reported previous infection with Covid-19	16 (50.0)	103 (27.5)	0.01
General trust in the principle of vaccination			<0.001
Totally agree/rather agree	26 (81.3)	356 (95.19)	
Neither agree nor disagree/rather disagree/totally disagree	6 (18.7)	18 (4.8)	
Willingness to get vaccinated against other diseases during fertility treatment (e.g., pertussis, influenza)			<0.001
Definitely yes/Rather yes	17 (53.1)	325 (86.9)	
Not sure/Rather no/definitely no	15 (46.9)	49 (13.1)	
Reported vaccination against influenza	4 (12.5)	91 (24.3)	0.13

Data are shown as mean ± standard deviation (SD) or number (%).

Of all participants 374 (92.1%) stated that they had been vaccinated against SARS-CoV-2 and 32 (7.9%) were not. Factors associated with the decision for vaccination were full time or part time employment (*p* = 0.05), a general high trust in the principle of vaccination (*p* < 0.001) and a high willingness for other vaccination during fertility treatment (*p* < 0.001) as well as risk factors for severe COVID-19 such as obesity or airway diseases (*p* = 0.01). In addition, unvaccinated participants were more likely to report an infection with COVID-19 in the past (*p* = 0.007).

### 3.2. Concerns and worries of the vaccinated group

**Figure 1:**
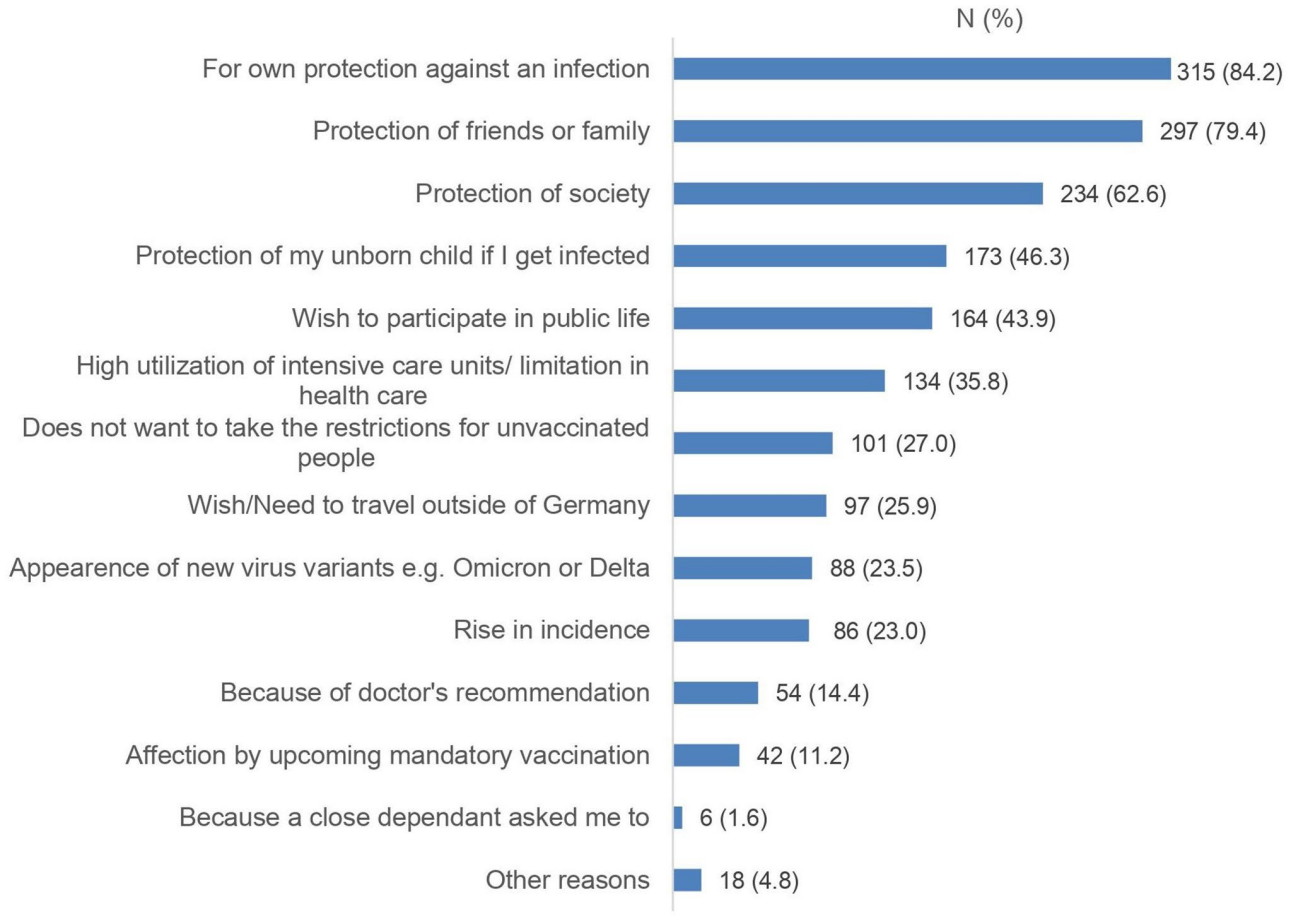
Reasons to decide for a SARS-CoV-2 vaccination in the vaccinated cohort (*N* = 374). Data are shown as numbers (%).

**Figure 2:**
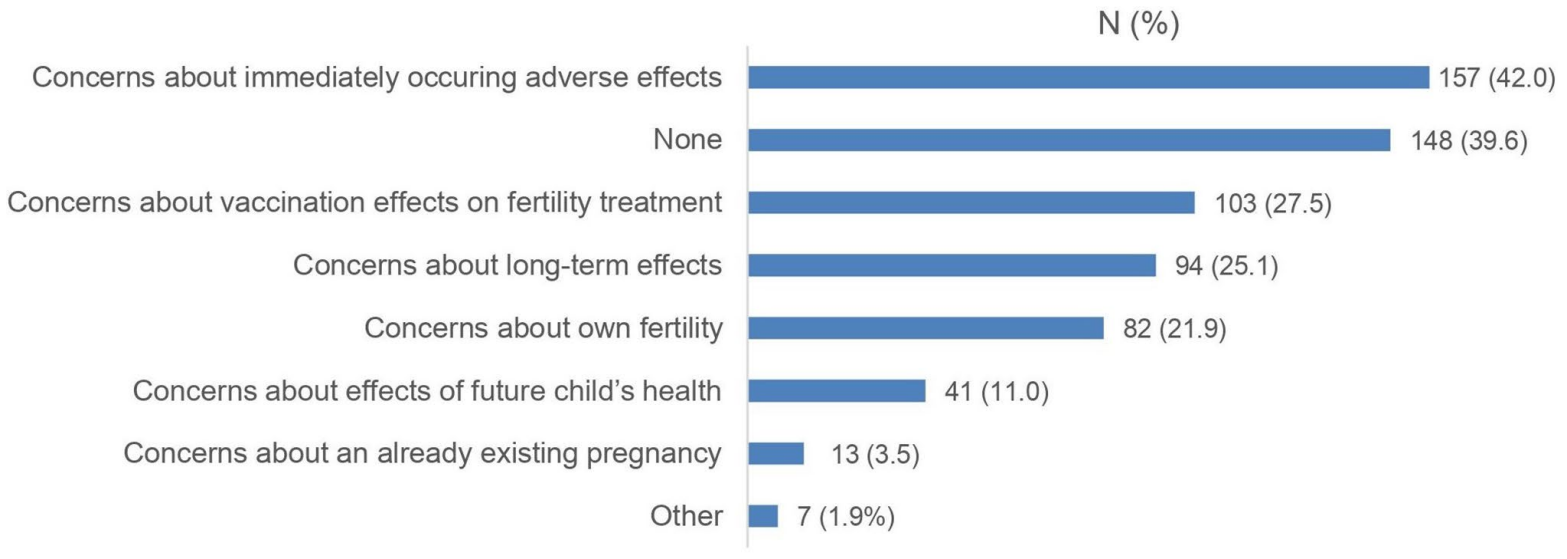
Concerns before a SARS-CoV-2 vaccination of the vaccinated cohort (*N* = 374). Data are shown as numbers (%).

The majority of vaccinated participants received three vaccine doses (*n* = 295, 78.9%). Most of them preferred the *Comirnaty vaccine* (Biontech/Pfizer; 60.7%), almost a quarter (22.2%) did not have a specific preference. In Figures 1, 2, reasons for getting vaccinated and previous concerns and worries of vaccinated participants are illustrated. While 39.6% of women had no concerns, about a quarter had concerns about their own fertility (21.9%) or impact on the fertility treatment (27.5%). Nevertheless, the most common concern was immediately occurring adverse effects after vaccination (42.0%).

**Figure 3:**
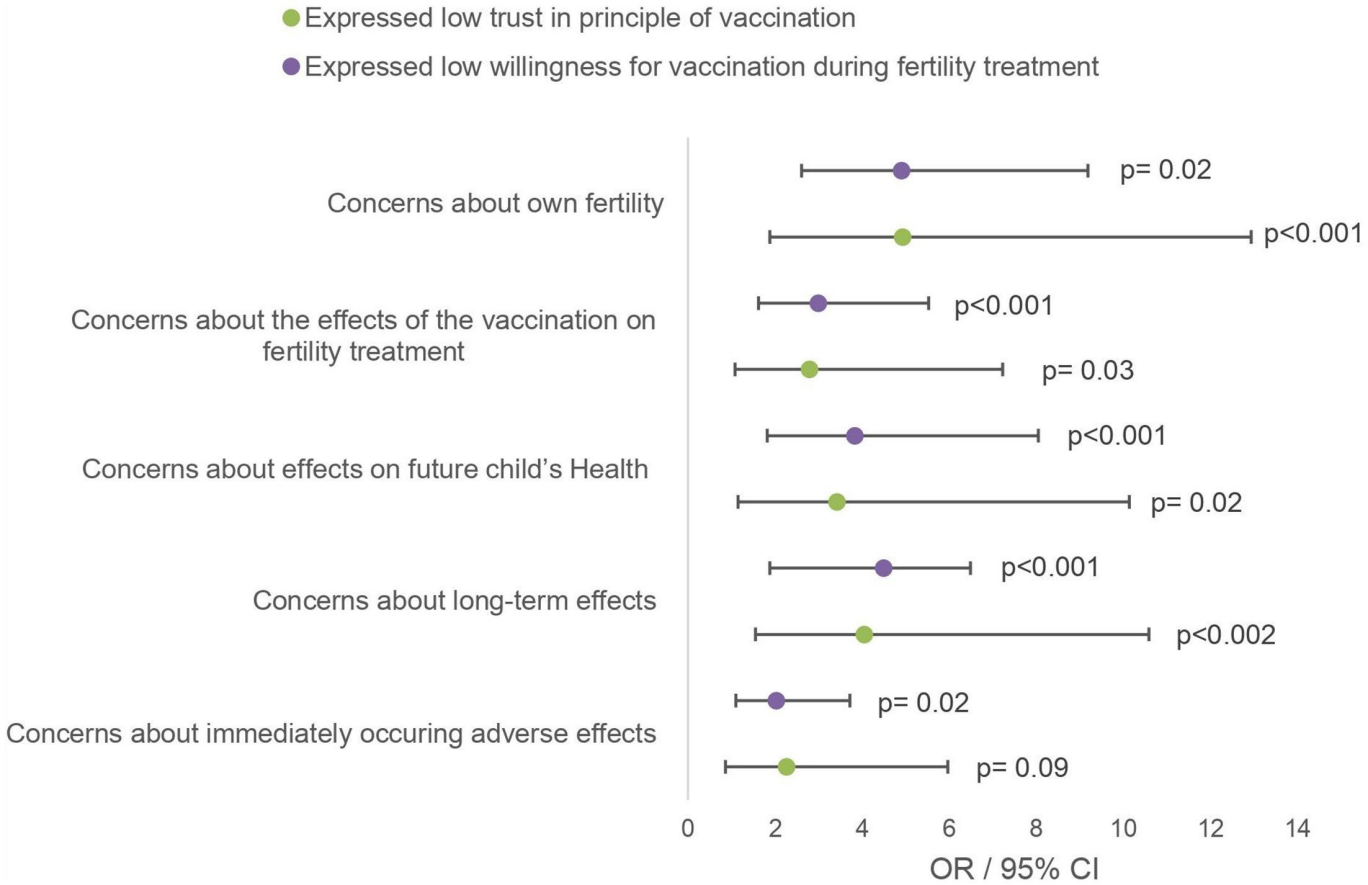
Bivariate analysis of health-related concerns of the vaccinated population. The given odds ratios relate to the group of participants who rated trust in principle of vaccination/willingness for other vaccination during fertility treatment as “high” or “rather high” on a 5-point Likert scale.

Figure 3 shows correlations between concerns prior to the vaccination and the general confidence in the principle of vaccination and willingness to vaccinate against other diseases during fertility treatment using odds ratios and their 95% confidence interval. The reported odds ratios refer to the individual risk of having had this concern before vaccination among participants with rather low confidence or low willingness to vaccinate during fertility treatment compared to high confidence or high willingness. The analysis revealed that participants with lower confidence had an increased risk of selecting the given worry. However, this effect was not significant for concerns about immediately occurring side effects (OR 2.26, 95% CI 0.86–5.97; *p* = 0.09). The described effect was even stronger in the group with low willingness to vaccinate during treatment. Not shown in the table but still worth mentioning is the association between low vaccination confidence and low willingness to vaccinate during treatment (OR 11.33, 95% CI 4.70–27.28, *p* < 0.001).

### 3.3. Attitudes and concerns of the unvaccinated group

When asked about detailed attitudes toward Sars-CoV-2 vaccination, 23 of the 32 (71.9%) unvaccinated participants completely rejected vaccination, 8 (12.5%) were still unsure, and only one person indicated that she is planning to get vaccinated soon.

**Figure 4:**
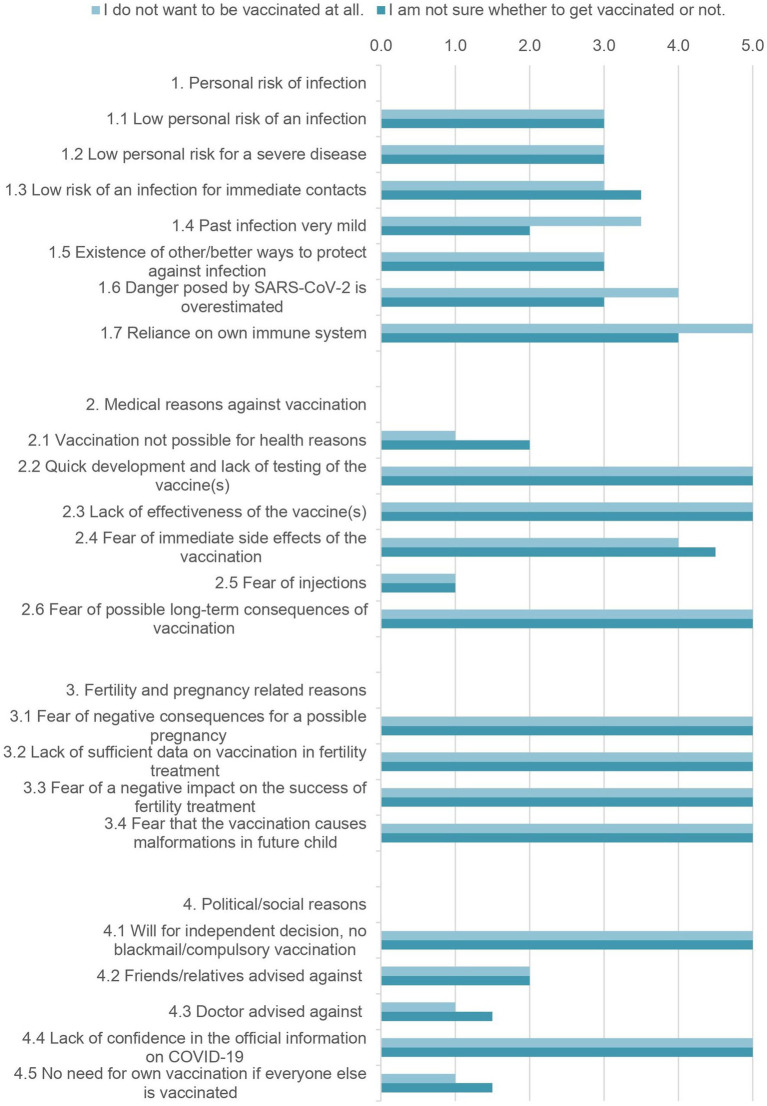
Detailed reasons against vaccination of the unvaccinated group (*N* = 32). The given values are the median ratings from 1 (=I do not agree at all) to 5 (=I totally agree) of the 5-point Likert scale.

Participants were asked to rate various statements regarding reasons against vaccination on a Likert scale. The statements to be rated and their median scores are shown in Figure 4, as well as the respective interquartile range, separately for the group of the undecided and the group of women who completely rejected the vaccination. For the sake of clarity, four categories were created. With regards to the personal risk for infection in the group that completely rejected vaccination, reliance on one’s own immune system (5.0 [4.0–5.0]) was the outstanding argument. Also highly rated (4.0 [3.0–4.0]) was the opinion that the risk posed by COVID-19 was overestimated.

In the field of medical reasons against vaccination the rapid development and lack of testing of vaccines (5.0 [4.0–5.0/5.0–5.0]) and too low efficacy (5.0 [4.0–5.0]) were highly rated. Fear of long-term consequences was equally high (5.0 [4.5–5.0/ 5.0–5.0]).

The third category asked for statements regarding fertility and the desire to have children. These were all rated by both groups with a median of 5.0 [4.0–5.0].

The last category dealt with social and political reasons. For both groups, the will to make an independent decision (5.0 [5.0–5.0]) and a lack of confidence in official information about COVID-19 (5.0 [3.75–5.0/4.25–5.0]) were the main reasons against a COVID-19 vaccination.

Not playing a major role in vaccination hesitancy for both groups were the fear of injections, advice from friends, family, or physicians, a medical contraindication, and a lack of need when everyone else is vaccinated.

The attitudes of declining and undecided participants showed no large discrepancies overall. Almost all unvaccinated participants, 31 (96.9%) stated they would not postpone their fertility treatment to get vaccinated. Five women (15.6%) would get vaccinated after successful fertility treatment and pregnancy, 8 (25.0%) were not sure and 19 (59.4%) would still refuse the vaccine. The emotional distress of the vaccination debate and the fertility treatment was rated very divided with a median of 3.5 [2.0–4.25].

Even though the results in Figure 4 give the impression that there was a need for more education, most of the unvaccinated participants refused further education for both infection with COVID-19 (81.3%) and the vaccination (88.0%).

## 4. Discussion

This study investigated the attitude of patients seeking fertility treatment toward the SARS-CoV-2 vaccination. While most participants (92.6%) in our cohort were vaccinated, uncertainties about the impact of vaccination on the infertility treatment and fertility were observed in both the vaccinated and unvaccinated group. Vaccinated individuals still showed worries about the vaccination correlated to general distrust in health care. The unvaccinated participants rated concerns about their fertility and infertility treatment as equal to general health concerns.

The unvaccinated rate of 7.4% is similar low as in other studies (13). Clinical experience suggests that the actual rate is even higher. The percentage of unvaccinated people in Germany is currently 22.1% (14). It can be assumed that a similar level is also found among infertile patients. Factors associated with being vaccinated were employment, high confidence in the general principle of vaccination, the presence of risk factors for a severe course of COVID-19, and a high willingness to have other vaccinations performed during fertility treatment. The role of employment can be explained by the introduction of mandatory vaccination for health care workers in Germany in 2022 (15). A US study has also shown that, for example, work colleagues can positively influence the vaccination decision (16). Surprisingly, no other factors such as educational level or age were associated with vaccination status, nor were treatment parameters or the stress of unfulfilled childbearing. However, it is well established that an unfulfilled desire to have children and fertility treatments are stressors in themselves (17). This has been exacerbated during the pandemic period by the temporary closure of fertility clinics (18) and may have indirectly influenced vaccination decisions. Although general confidence in vaccination was relatively high in this study population, the association of mistrust and unvaccination confirms the findings of previous studies (13). Latest findings have also shown a connection between medical mistrust and COVID-19 vaccine hesitancy in women undergoing fertility treatment (13). Low willingness to receive vaccination during fertility treatment was associated with low general trust in vaccination, pointing out the connection between infertility and vaccine hesitance in our study as well.

This is consistent with the detailed survey and analysis of vaccinated participants. Their motives for vaccination were not clearly related to the desire to have children, but rather their own protection was the decisive factor. Fear of negative effects on fertility, fertility treatment and pregnancy indicate that further education is still needed even among vaccinated patients. The association of these worries with lower confidence in vaccination and low willingness to vaccinate is in line with an US American study that investigated the association between medical mistrust and vaccine hesitancy (13). According to a 2019 WHO statement, vaccine hesitancy is one of 10 threats to global health (19). Multiple studies report an increasing vaccine hesitance since the COVID-19 pandemic in several countries (20).

In the group of unvaccinated women, the decisive reasons against vaccination are similar to those in the rest of the population, as was found out in a survey in Germany in 2021 (21). Here, fear of side effects, too brief clinical testing before the introduction of the vaccines and the desire for independent decision making and without blackmail or compulsory vaccination were most frequently mentioned (21). These reasons were also rated as most important and decision driving by our study population. However, in contrast to the general population, fears regarding a negative impact on fertility and pregnancy were rated equally high. Somewhat contradictory was the rejection of additional education for both SARS-CoV-2 infection and COVID-19 vaccination of most of the unvaccinated respondents in our study. It can be assumed that this attitude is caused by mistrust in the health care system and its representatives. This is supported by a US study that found significant associations between mistrust in health care and underutilization of health care services, such as counseling and education (22). It was also confirmed by a more recent study performed during the COVID-19 pandemic (23). Thus, this rejection of more education in the context of the participants’ views is a central finding of our work that should not be ignored.

Our survey also showed that almost all unvaccinated participants would not postpone their treatment to be vaccinated beforehand. Decline of fertility over time and inferior outcome of an assisted reproductive technique (ART) treatment were serious concerns of women during the first wave of the pandemic when fertility clinics stopped their treatments (18). Here, an Italian study provided reassuring data and found no impact of postponement of treatment during the COVID-19 pandemic on reproductive outcomes of women utilizing fertility treatment (24). Nevertheless, pressure and anxiety about the “biological clock” was increasing in a United Kingdom study performed during the pandemic, and respondents stated that they still lacked education about general fertility decline with increasing age (25).

In our study, both vaccinated and unvaccinated women stated fertility concerns. A Chinese study has shown that up to 2 months after the application of an inactivated SARS-CoV-2 vaccine a reduced pregnancy rate can result after IVF treatments. From day 61 onward, this effect decreased, and from day 91 onward, it was no longer observed (26). The authors conclude that consequently, a short pause of the therapy would be quite reasonable. Another study from Israel did not find any negative effects of vaccination on fertility parameters, e.g., Anti-Muellerian Hormone (AMH) concentrations in vaccinated women undergoing IVF (27). Latest retrospective analyses of IVF cycles have also not demonstrated a lower pregnancy rate associated with the COVID-vaccination (28–30). To date, however, there have been no studies showing that COVID-19 vaccination can affect fertility in the long term. This was reported by several analyses from different countries (31, 32). Nevertheless, further studies are needed for a long-term evaluation.

To our knowledge, this study is the first to explore detailed fears and concerns of both vaccinated and unvaccinated people. We believe that it is very important to clarify not only the concerns of the unvaccinated, but also those of the vaccinated, to ensure and maintain trust and compliance. It is also the first study of its type in Germany and was conducted as a multi-center study across the country. Due to this, we were able to avoid bias with regards to any regional differences. Nevertheless, most respondents came from two federal states (Lower Saxony and North Rhine-Westphalia) owing to an active and not just passive recruitment by the respective fertility care providers. However, due to the anonymous character of the survey we are not able to specify the fertility clinic.

Another strength of this study is the high response rate. Of 981 people who clicked on the link, 485 began answering the questionnaire and 406 finished the survey, giving a response rate of 49.4% and a completion rate of 83.7%. This is even higher than in comparable studies (13, 33), and can also be explained by the fact that double clicks on the survey link were counted as well as people who first only looked at the questionnaire, and then answered it later.

Our study also has limitations. Although we were planning to include the partner perspectives in our analysis this was not possible. During the study period there was a persistent limited access to medical facilities for persons other than the immediate patient. It was impossible for us to approach the partners directly and we decided to focus only on the immediate patient. Therefore, the vaccination status and attitudes toward a SARS-CoV2-vaccination of the partners remains unknown and should be the focus of future studies.

Our study might also have a selection bias because of the overrepresentation of highly educated women. In general, patients with high medical mistrust might not be as willing to take part in an academic study. Thus, we might not have pictured the opinion of extreme conspiracy theorists in our study which makes it even harder to reach out for this specific group. In future research ways of including them are to be set up.

In addition, although we included questions regarding the preference of a vaccine, most of the questions generally referred to “the vaccine(s).” Since the Comirnaty vaccine (Biontech/Pfizer) is the first and main vaccine used in Germany (34), the results are probably most applicable to this compound.

The couples who were contacted were all most likely undergoing treatment during different stages of the pandemic. Transferring the results to all infertile couples should therefore be done with caution. Due to the collection of exclusively subjective perceptions, false positive results cannot be excluded.

Overall, our study found trends of mistrust and fear of side effects on fertility of the COVID-19 vaccination in both vaccinated and unvaccinated patients seeking infertility treatment. To date, those effects on fertility have not been proven so far. Still, every patient’s concern, no matter if vaccinated or not has to be taken seriously to avoid medical mistrust and additional emotional distress during fertility treatment.

## Data availability statement

The raw data supporting the conclusions of this article will be made available by the authors, without undue reservation.

## Ethics statement

The studies involving human participants were reviewed and approved by the Ehtics Committee of Hannover Medical School. The patients/participants provided their written informed consent to participate in this study.

## Author contributions

JK: data collection, data analysis, and manuscript writing. CS: project development and manuscript editing. DF: manuscript editing. AB: project development and manuscript editing. FV-H: project development and protocol, data collection, and manuscript editing. All authors contributed to the article and approved the submitted version.

## Funding

This study was supported by departmental funds of the Department of Obstetrics, Gynecology and Reproductive Medicine, Hannover Medical School, Germany.

## Conflict of interest

The authors declare that the research was conducted in the absence of any commercial or financial relationships that could be construed as a potential conflict of interest.

## Publisher’s note

All claims expressed in this article are solely those of the authors and do not necessarily represent those of their affiliated organizations, or those of the publisher, the editors and the reviewers. Any product that may be evaluated in this article, or claim that may be made by its manufacturer, is not guaranteed or endorsed by the publisher.
