# Supplementary material for: Fear of fertility side effects is a major cause for COVID-19 vaccine hesitance in infertile patients
Source: Front Med (Lausanne). 2023 Jun 1;10:1178872. doi: 10.3389/fmed.2023.1178872 (PMC10267368; doi:10.3389/fmed.2023.1178872)
Supplement: Supplementary file 1 [file Table_1.DOCX]

Supplemental Table 1

| State/Region | N (%) |
| --- | --- |
| Baden-Wuerttemberg | 39 (9.6) |
| Bavaria | 45 (11.1) |
| Berlin | 10 (2.5) |
| Brandenburg | 12 (3.0) |
| Bremen | 8 (2.0) |
| Hamburg | 3 (0.7) |
| Hesse | 18 (4.4) |
| Mecklenburg-Western Pomerania | 3 (0.7) |
| Lower Saxony | 125 (30.8) |
| North Rhine-Westphalia | 79 (19.5) |
| Rhineland-Palatinate | 8 (2.0) |
| Saarland | 2 (0.5) |
| Saxony | 8 (2.0) |
| Saxony-Anhalt | 10 (2.5) |
| Schleswig Holstein | 6 (1.5) |
| Thuringia | 7 (1.7) |
| Outside Germany | 23 (5.7) |
