# Supplementary material for: Fear of fertility side effects is a major cause for COVID-19 vaccine hesitance in infertile patients
Source: Front Med (Lausanne). 2023 Jun 1;10:1178872. doi: 10.3389/fmed.2023.1178872 (PMC10267368; doi:10.3389/fmed.2023.1178872)
Supplement: Supplementary file 2 [file Data_Sheet_1.docx]

**Original survey translated into English language**

**Title: Fear of fertility side effects is a major cause for Covid-19 vaccine hesitance in infertile patients**

| **Introducing Question** |
| --- |

**Are you yourself** undergoing infertility treatment **or are you the partner** of **person undergoing infertility treatment**?

- I am planning an infertility treatment
- I am currently undergoing infertility treatment.
- I am a partner of a patient who is planning infertility treatment.
- I am the partner of a patient who is currently undergoing infertility treatment.

| **General Information** |
| --- |

**1. Age*:***___

**2. What is the highest level of education you have achieved:**

- Finished school without graduation
- Still at school
- Secondary school leaving certificate
- Secondary school certificate
- Vocational baccalaureate
- General higher education entrance qualification (Abitur)
- Completed vocational training
- University Degree
- Higher academic qualification (doctorate/habilitation)

**3. What is your current job?**

- employed full-time
- employed part-time
- seeking work
- self-employed
- on parental leave
- on leave/unable to work
- at school
- in university
- in vocational education and training
- Other: __­­­ __

**4. In which federal state do you live?**

- Baden-Wuerttemberg
- Bavaria
- Berlin
- Brandenburg
- Bremen
- Hamburg
- Hesse
- Mecklenburg-Western Pomerania
- Lower Saxony
- North Rhine-Westphalia
- Rhineland-Palatinate
- the Saarland
- Saxony
- Saxony-Anhalt
- Schleswig-Holstein
- Thuringia
- I do not live in Germany.

| **Previous pregnancies/fertility treatment** |
| --- |

**5. I've been pregnant before.** **^1^**

- yes
- no

*If yes:*

*Number of pregnancies:_____*

*therefrom:*

- *Number of live births:___*
- *Number of miscarriages:___*
- *Number of abortions:____*
- *Number of ectopic pregnancies: ____*

^1^Question only for female participants

**6. I am undergoing fertility treatment^2^**

- physically involved (my sperm is used)
- not physically involved

^2^Question only for the partners

**7.**  **The unfulfilled desire to have children has existed since...**

- less than 1 year
- more than 1 and less than 2 years
- more than 2 and less than 5 years
- more than 5 years

**8.**  **The psychological burden of the unfulfilled desire to have children and any associated treatments I feel as ...**

- very high
- rather high
- neutral
- rather low
- very low

**9.** I **have already had** **the following treatments**:

- - Sexual intercourse at the optimal time
  - Intrauterine insemination
  - in vitro fertilization or intracytoplasmatic sperm injection (IVF/ICSI) with embryo transfer
  - Frozen embryo transfer
  - None
  - Other:_____

| **COVID-19** |
| --- |

**10.**  **I have witnessed a friend or close relative become seriously ill with Covid-19.**

- Yes
- No

**11.**  **I have lost a friend or close relative to Covid-19.**

- Yes
- No

**12.**  **I work in a SARS-CoV-2 risk area (e.g. Health Care)**

- Yes
- No

**13.**  **Do you have risk factors for a severe course of SARS-CoV-2 infection? Please tick the appropriate box!**  **Multiple answers are possible.**

- Obesity
- Diabetes mellitus
- Chronic hypertension
- Respiratory diseases
- Cancer
- Cardiovascular disease
- Liver disease
- Kidney disease
- Autoimmune disease
- Other (please explain):
- I have no risk factors.

**14.**  **Basically**, **I trust the principle of vaccination against the common diseases (measles, mumps, rubella, etc.).**

- I totally agree.
- I rather agree.
- Neither agree nor disagree.
- I rather disagree.
- I totally disagree.

**15.**  **If medically recommended, I would have the usual vaccinations refreshed**  or carried out **during fertility treatment. (e.g. pertussis, flu)**

- I totally agree.
- I rather agree.
- Neither agree nor disagree.
- I rather disagree.
- I totally disagree.

**16.**  **I am vaccinated against influenza (flu):**

- Yes
- No

**17.**  **I have already had an infection with SARS-CoV-2:**

- Yes
- No

**If SARS-CoV-2 vaccination YES:**

**18.**  **I am:**

- Simply vaccinated
- Double vaccinated
- Triple-vaccinated (boosted)
- Quadruple vaccinated (2nd booster)
- Recovered from SARS-CoV-2 infection and received appropriate booster
- Other (please explain):

**19.**  **What were the decisive reasons for you to carry out the vaccination?**

- Own protection against infection
- Protection of my unborn child if I get ill
- Protection of relatives, friends
- Protection of the general population
- Because I want to participate in public life.
- Because I also want to travel outside Germany
- Because I do not want to accept the restrictions for unvaccinated people
- Since I am/will be affected by the compulsory vaccination for certain occupational groups
- The increase in incidences.
- The emergence of new virus variants (e.g. Delta, Omicron)
- The high occupancy of intensive care units or restrictions on medical care
- Because a close relative asked me to
- Because my doctor advised me to
- Other (please explain):

**20.**  **Before carrying out the vaccination, I had**

- No worries
- Concerns about immediate side effects
- Concerns about long-term effects
- Concerns about a pre-existing pregnancy
- Concerns about my fertility
- Concerns about the effects of vaccination on fertility treatment
- Concerns about the effects of vaccination on the health of my future fetus
- Other (please explain):

**21.**  **I had a preference for the vaccine before** **getting vaccinated**:

- No, I didn't care about a specific vaccine.
- Yes, I **didn't** want to be vaccinated with an mRNA vaccine-
- Yes, I **wanted to** be vaccinated with *Comirnaty* (Biontech/Pfizer (Comirnaty, mRNA vaccine).
- Yes, I **didn't** want to be vaccinated with *Vaxzevria* (Astra Zeneca).
- Yes, I **didn't** want to be vaccinated *with* **COVID-19** *Vaccine Janssen* (Johnson & Johnson).
- Other preferences: ___

(Here end of the questionnaire for COVID-19 vaccination "Yes" and go to the final question)

**If no COVID-19 vaccination so far**

**22.**  **Attitude towards vaccination**

- I plan to get vaccinated against SARS-CoV-2 in the future.
- I am undecided whether to get vaccinated.
- I will definitely not get vaccinated.

**23.**  **I haven't been vaccinated against SARS-CoV-2 (**yet) because**...**

|  | Fully true | Partly true | neutral | Rather not true | Not true at all |
| --- | --- | --- | --- | --- | --- |
| ... the risk of infection is not high for me. |  |  |  |  |  |
| ... the risk for me to become seriously ill is not high. |  |  |  |  |  |
| ... the risk of infection for my immediate contacts is not high. |  |  |  |  |  |
| ... I have already had a Sars-CoV-2 infection which has been very mild. |  |  |  |  |  |
| ... there are other/better means to protect against the COVID-19 infection. |  |  |  |  |  |
| ... I rely on my own immune system. |  |  |  |  |  |
| ... the vaccine(s) have been developed so quickly and have not been tested long enough. |  |  |  |  |  |
| ... I want to decide independently, without blackmail/compulsory vaccination. |  |  |  |  |  |
| **...** I have a lack of confidence in the official information on COVID-19. |  |  |  |  |  |
| ... I am afraid of the immediate side effects of the vaccination. |  |  |  |  |  |
| ... I am afraid of injections. |  |  |  |  |  |
| ... I am afraid of the possible long-term effects of the vaccination. |  |  |  |  |  |
| ... I am concerned that the vaccination has negative consequences for a possible pregnancy. |  |  |  |  |  |
| ... there is a lack of sufficient data on vaccination in fertility treatment. |  |  |  |  |  |
| ... I am concerned that vaccination could have a negative impact on the success of fertility treatment. |  |  |  |  |  |
| ... I am afraid that the vaccination could lead to malformations in my child. |  |  |  |  |  |
| ... I do not consider the vaccines to be effective enough. |  |  |  |  |  |
| ... I think the danger posed by SARS-COV-2 is overestimated. |  |  |  |  |  |
| ... my friends/relatives have advised against it. |  |  |  |  |  |
| ... my doctor advised against it. |  |  |  |  |  |
| ... I am not allowed to be vaccinated for health reasons. |  |  |  |  |  |
| ... I think that if everyone is vaccinated, I don't have to get vaccinated. |  |  |  |  |  |

**Other reasons: __**__

(Please explain.)

**24.**  **To decide to get vaccinated,**

- I would like to see more information about vaccination from the media
- I would like to get more information about vaccination by my family doctor
- I would like to get more information about vaccination by my gynecologist
- I would like to get more information about vaccination by my fertility doctor
- more education does not help me either.

**25.**  **To decide to get vaccinated,**

- I would like to see more media education about the consequences of infection with COVID-19
- I would like to get more information about the consequences of infection with COVID-19 from my family doctor
- I would like to get more information about the consequences of infection with COVID-19 by my gynaecologist
- I would like to get more information about the consequences of infection with COVID-19 by my fertility doctor
- more education about the consequences of an infection does not help me either.

**26.**  **If more education is wished: I would like to get more information about**

- the impact of vaccination on my immediate health.
- the impact of vaccination on my long-term health.
- the impact of vaccination on my fertility (long-term).
- the impact of vaccination on my partner's fertility.
- the influence of vaccination on the success of fertility treatment (current, short-term)
- the influence of vaccination on pregnancy.

**27. If more education is wished: I would like to see more information about**

- the impact of a COVID-19 infection on my fertility (long-term)
- the impact of COVID-19 infection on pregnancy.
- the impact of a COVID-19 infection on my immediate health.
- the impact of COVID-19 infection on my long-term health.
- The influence of a COVID-19 infection on the success of fertility treatment (current, short-term)

**28.**  **I would get vaccinated against SARS-CoV-2 with the following vaccine (names of vaccines in italics):**

- Biontech *Comirnaty* (mRNA vaccine)
- Moderna *Spikevax* (mRNA vaccine)
- Astra Zeneca *Vaxzevria* (vector vaccine)
- Johnson & Johnson *Janssen* (vector vaccine)
- Novavax *Nuvaxovid* (lethal vaccine) /protein vaccine
- I don't care about the vaccine.
- I wouldn't get vaccinated with any vaccine.
- Other:

**29.**  **I would postpone my fertility treatment in order to get vaccinated beforehand:**

- Yes
- No
- I don't know

**30.**  **I will only be vaccinated after successful fertility treatment and completion of pregnancy:**

- Yes
- No
- I don't know yet

**31.**  **Against the background of my fertility treatment, the vaccination debate is a psychological burden.**

- Very strong
- Rather strong
- Neural
- Rather low
- Very low

**32.**  **I am informing myself about the COVID-19 vaccination...**

- On the Internet
- Via social media (Youtube, Instagram, Facebook)
- at others (e.g. friends/relatives/colleagues)
- By reading scientific studies
- at my doctor
- Not at all
- Others (please specify):

**33.**  **If I get infected with SARS-CoV-2,**

|  | Totally agree | Tend to agree | Neither agree nor disagree | Tend to disagree | Totally disagree |
| --- | --- | --- | --- | --- | --- |
| I am worried about my own health |  |  |  |  |  |
| I am worried about the health of family members |  |  |  |  |  |
| I am worried about my fertility |  |  |  |  |  |
| I am worried about a possible pregnancy |  |  |  |  |  |

**More:_____**

**34.**  **The following factors make me rethink my attitude...**

|  | Fully | Partly | Neutral | Rather not | Not at all |
| --- | --- | --- | --- | --- | --- |
| Information on increasing incidences |  |  |  |  |  |
| New virus variants (e.g. Omicron) |  |  |  |  |  |
| Information on overcrowded hospitals/restrictions on medical care |  |  |  |  |  |
| Information on a possible upcoming vaccination obligation |  |  |  |  |  |
| Current mandatory restrictions for unvaccinated people |  |  |  |  |  |

More:________

**35. Do you have any other comments? If so, which ones?**
